# Function and Mechanism of Antiviral Wasp Venom Peptide Protopolybia-MP III and Its Derivatives against HSV-1

**DOI:** 10.3390/toxins16030132

**Published:** 2024-03-04

**Authors:** Fang Sun, Xiangdong Ye, Tanran Han, Jingwen Xia, Lili Wu, Wen Zhu, Li Ding, Xudong Luo, Chenhu Qin, Zongyun Chen

**Affiliations:** 1 Department of Biochemistry and Molecular Biology, Institute of Basic Medical Sciences, College of Basic Medicine, Hubei Key Laboratory of Embryonic Stem Cell Research, Hubei University of Medicine, Shiyan 442000, China; 2016202040055@whu.edu.cn (F.S.); yexiangdong7237@163.com (X.Y.); tanranhan2032@163.com (T.H.); xiajingwen1214@163.com (J.X.); wulili31926@163.com (L.W.); zhuwen0712@126.com (W.Z.); luoxudong000@126.com (X.L.); 2016202040025@whu.edu.cn (C.Q.); 2 Hubei Key Laboratory of Wudang Local Chinese Medicine Research, Hubei University of Medicine, Shiyan 442000, China; 3 Department of Clinical Laboratory, Dongfeng Hospital, Hubei University of Medicine, Shiyan 442000, China; dl2168@163.com

**Keywords:** wasp venom peptide, Protopolybia-MP III, HSV-1, antiviral peptide design

## Abstract

Viruses are one of the leading causes of human disease, and many highly pathogenic viruses still have no specific treatment drugs. Therefore, producing new antiviral drugs is an urgent matter. In our study, we first found that the natural wasp venom peptide Protopolybia-MP III had a significant inhibitory effect on herpes simplex virus type 1 (HSV-1) replication *in vitro* by using quantitative real-time PCR (qPCR), Western blotting, and plaque-forming assays. Immunofluorescence analysis showed Protopolybia-MP III could enter cells, and it inhibited multiple stages of the HSV-1 life cycle, including the attachment, entry/fusion, and post-entry stages. Furthermore, ultracentrifugation and electron microscopy detected that Protopolybia-MP III significantly suppressed HSV-1 virion infectivity at different temperatures by destroying the integrity of the HSV-1 virion. Finally, by comparing the antiviral activity of Protopolybia-MP III and its mutants, a series of peptides with better anti-HSV-1 activity were identified. Overall, this work found the function and mechanism of the antiviral wasp venom peptide Protopolybia-MP III and its derivatives against HSV-1 and laid the foundation for the research and development of wasp venom-derived antiviral candidate peptide drugs.

## 1. Introduction

Since 2010, the World Health Organization has declared 28 health emergencies, 15 of which were viral outbreaks (https://www.who.int/emergencies/situations) (accessed on 29 December 2023). Viruses are one of the leading causes of human disease, as the discovery and development of new vaccines are often both challenging and time-consuming [1]. The most commonly used alternative method for viral control in clinical practice is treatment with antiviral drugs, such as ribavirin, aciclovir, and polyinosinic-polycytidylic acid [2,3]. But many highly pathogenic viruses still have no specific treatment drugs [4]. In addition, with the increase in viral resistance and the prevalence of new viruses, there is an urgent need to produce new antiviral drugs.

Recent evidence has shown that some peptides derived from animals, plants, or microorganisms also present broad-spectrum antiviral activity, called antiviral peptides (AVPs) [5,6,7]. Various mechanisms of antiviral action of AVPs have been found. AVPs can act as viricides that directly target virions to exert antiviral activity, such as the amphibian antimicrobial peptide Temporin L [8]. Some target infected cells and exert antiviral effects at various stages of the viral life cycle by binding viral proteins or nucleic acids and regulating the activity of host factors or signaling pathways that play important roles in the process of viral replication, such as the scorpion venom peptide Ev37 [9]. Some, like human cathelicidin LL-37, are immunomodulators targeting the host immune system and cells to promote the expression of immune-related genes such as interferon, thereby inducing the production of interferon-stimulating genes to inhibit viral replication [10]. AVPs have the advantages of high efficiency, high specificity, and good safety, and they represent an important direction for the development of new antiviral drugs [11,12]. Therefore, it is of great significance to mine new AVPs and explore their antiviral mechanisms.

Wasps are a widely distributed and diverse group of insects whose venom has natural medicinal properties, including suppressing inflammation and analgesia, improving blood circulation, improving heart function, and immunosuppressing [13,14]. Wasp venom contains a variety of bioactive substances, among which Mastoparan family peptides are a class of small-molecule peptides with the highest content and the largest number of members in wasp venom [15,16]. Previous studies have shown that Mastoparan family peptides are an important class of lead drug peptide molecules that have various pharmacological effects, such as inhibiting bacterial infection, regulating the immune system, and providing antitumor activity [16,17,18]. However, the antiviral functions of natural wasp venom Mastoparan family peptides are still unknown.

HSV-1 is a double-stranded, enveloped DNA virus, and it infects more than 60% of the world’s population [19]. HSV-1 is a widely distributed neurophilic human pathogen that remains in the host for their lifetime after infection and is highly contagious; it clinically manifests as localized herpes of the mucous membrane or skin and can cause diseases such as oral gingivitis, herpes encephalitis, and herpetic keratitis [20,21,22]. Clinically, drugs used to treat HSV-1 infection include acyclovir (ACV) and its analogues, which work by preventing the synthesis of viral DNA, but this class of drugs can develop resistance and fail to eliminate the existing virus and regulate its storage in the trigeminal ganglion [23]. Currently, there is no vaccine to prevent or treat HSV-1 infection, and no drug can completely prevent HSV-1 recurrence [23]. Therefore, it is of great value to search for anti-HSV-1 drug candidates with good specificity and high activity.

Here, the antiviral activity and mechanism of Protopolybia-MP III, which is derived from the venom of the neotropical social wasp *Protopolybia exigua* of the *Polybiidae* family, were systematically analyzed. First, the antiviral activity of Protopolybia-MP III in vitro was examined by qPCR, Western blotting, and plaque formation assays. The results indicated that Protopolybia-MP III strongly inhibited HSV-1 replication in Vero cells, A549 cells, human corneal epithelial cells (HCEC), and human neuroepithelial cells (SK-N-MC). Next, the antiviral action stage of Protopolybia-MP III was determined, and the results showed that Protopolybia-MP III inhibited the attachment, entry/fusion, and post-entry stages of the HSV-1 life cycle. Furthermore, immunofluorescence showed that Protopolybia-MP III entered Vero cells and decreased the expression of HSV-1 ICP0. In addition, the influence of Protopolybia-MP III on HSV-1 free virion infectivity was analyzed, and we found that Protopolybia-MP III significantly inhibited HSV-1 virion infectivity at 4 °C, 22 °C, and 37 °C. Moreover, it was determined that Protopolybia-MP III was able to destroy the integrity of the HSV-1 virion using ultracentrifugation and electron microscopy. Finally, the antiviral activity residues of Protopolybia-MP III were determined using a point mutation technique, and Protopolybia-MP III mutants with higher anti-HSV-1 activity were obtained.

## 2. Results

### 2.1. Protopolybia-MP III Strongly Inhibits HSV-1 Replication In Vitro

**Figure 1:**
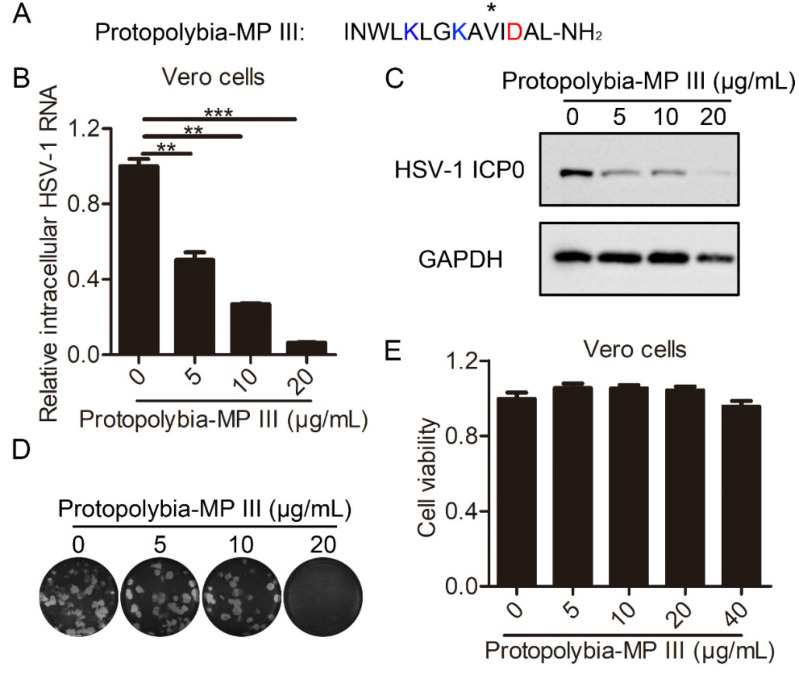
Protopolybia-MP III strongly inhibits HSV-1 replication in Vero cells. (**A**) Amino acid sequence of Protopolybia-MP III. Basic residues are labeled blue, and acidic residues are labeled red. (**B**,**C**) Suppression of intracellular HSV-1 infection by Protopolybia-MP III. Cells were preincubated with Protopolybia-MP III at different concentrations or PBS for 1 h and then infected with HSV-1 at a multiplicity of infection (MOI) of 1. After 24 h, the cells were collected. Intracellular HSV-1 RNA was analyzed by qPCR (**B**), and intracellular HSV-1 ICP0 protein levels were analyzed by Western blotting (**C**). (**D**) Suppression of Protopolybia-MP III on extracellular HSV-1 particles. Cells were treated with Protopolybia-MP III at different concentrations or PBS for 24 h, and then the supernatant was collected and evaluated in a plaque formation assay. (**E**) Cytotoxicity of Protopolybia-MP III to Vero cells. Cells were treated with Protopolybia-MP III or PBS at different concentrations for 24 h, and then the cytotoxicity was determined by CCK8 assay. * *p* < 0.05. ** *p* < 0.01. *** *p* < 0.001.

Protopolybia-MP III, derived from wasp venom, consists of 14 amino acids and has an amino modification at the carboxyl (C) terminus (Figure 1A). To determine the antiviral activity of Protopolybia-MP III against HSV-1 infection, the effects of different concentrations of Protopolybia-MP III on HSV-1 replication were detected in HSV-1-susceptible Vero cells [24]. The qPCR results showed that Protopolybia-MP III suppressed HSV-1 RNA in a concentration-dependent manner (Figure 1B). At Protopolybia-MP III concentrations of 5, 10, and 20 μg/mL, the inhibition rates of Protopolybia-MP III on intracellular HSV-1 RNA were 50%, 73%, and 94%, respectively. The 50% inhibitory concentration (IC_50_) of Protopolybia-MP III against HSV-1 RNA is 5.132 ± 1.054 μg/mL. A Western blotting assay showed that the intracellular HSV-1 ICP0 expression level was also inhibited by Protopolybia-MP III in a concentration-dependent manner (Figure 1C). At Protopolybia-MP III concentrations of 5, 10, and 20 μg/mL, the inhibition rates of Protopolybia-MP III on the intracellular HSV-1 ICP0 expression level were 67%, 74%, and 96%, respectively, as calculated by ImageJ software. In addition, we found that fewer plaques were produced when infected cells were treated with Protopolybia-MP III than without Protopolybia-MP III treatment (Figure 1D), suggesting that the extracellular supernatant treated with Protopolybia-MP III has fewer HSV-1 particles than that treated without Protopolybia-MP III. Then, the cytotoxicity of Protopolybia-MP III to Vero cells was determined using the CCK-8 assay. Protopolybia-MP III, at a concentration of 40 μg/mL, was almost nontoxic to Vero cells (Figure 1E). Overall, these results indicate that Protopolybia-MP III inhibits HSV-1 replication in Vero cells in a concentration-dependent manner.

**Figure 2:**
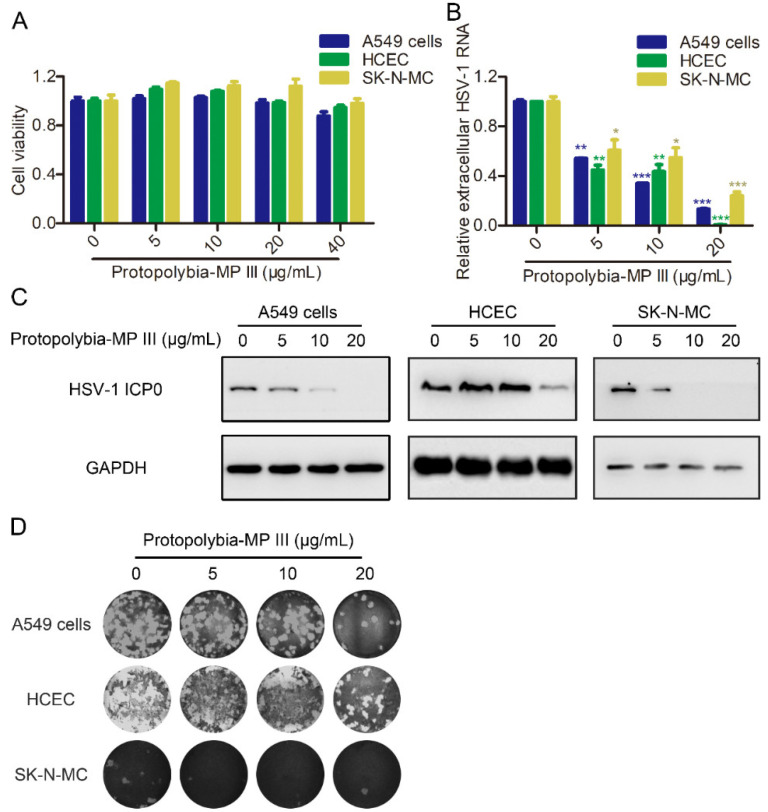
Protopolybia-MP III inhibits HSV-1 replication in human cell lines. (**A**) Cytotoxicities of Protopolybia-MP III to A549 cells, HCEC, and SK-N-MC. Cells were treated with Protopolybia-MP III at different concentrations or PBS for 24 h, and then cytotoxicities were determined by the CCK8 assay. (**B**,**C**) Suppression of intracellular HSV-1 infection by Protopolybia-MP III in A549 cells, HCEC, and SK-N-MC. Cells were preincubated with Protopolybia-MP III at different concentrations or PBS for 1 h and then infected with HSV-1 at an MOI of 1. After 24 h, the cells were collected. Intracellular HSV-1 RNA was analyzed by qPCR (**B**), and intracellular HSV-1 ICP0 protein expression levels were analyzed by Western blotting (**C**). (**D**) Inhibition of Protopolybia-MP III on extracellular HSV-1 particles in A549 cells, HCEC, and SK-N-MC. Cells were treated with Protopolybia-MP III at different concentrations or PBS for 24 h, and then the supernatant was collected and evaluated in a plaque formation assay. * *p* < 0.05. ** *p* < 0.01. *** *p* < 0.001.

To further clarify the antiviral activity of Protopolybia-MP III, we examined its effect on HSV-1 replication in other human cell lines, including HSV-1-susceptible A549 cells, HCEC, and neural-derived SK-N-MC [25,26,27]. Because the primary infection sites of HSV-1 mainly include epithelial tissues, while sensory neurons represent the main sites of the latent infection [20]. First, the cytotoxicities of Protopolybia-MP III to A549 cells, HCEC, and SK-N-MC were examined. The CCK8 assay showed that Protopolybia-MP III at a concentration of 40 μg/mL was almost nontoxic to A549 cells, HCEC, and SK-N-MC (Figure 2A). Then, the antiviral activity of Protopolybia-MP III was determined in these cell lines. The results showed that Protopolybia-MP III suppressed HSV-1 RNA in A549 cells, HCEC, and SK-N-MC in a concentration-dependent manner (Figure 2B). In addition, Western blotting assays showed that intracellular HSV-1 ICP0 expression levels were strongly inhibited by Protopolybia-MP III in these cell lines (Figure 2C). Similarly, we found that extracellular HSV-1 levels were lower in A549 cells, HCEC, and SK-N-MC treated with Protopolybia-MP III than in those treated with phosphate-buffered saline (PBS) by plaque formation assay (Figure 2D). By comparing the antiviral effects of Protopolybia-MP III at the RNA level, protein level, and extracellular supernatant level in four cell lines, we found that Protopolybia-MP III had a well-established inhibitory effect against HSV-1 in different cell lines, suggesting that Protopolyb-ia-MP III was a good AVP. In summary, these results indicate that Protopolybia-MP III strongly inhibits HSV-1 replication *in vitro*.

### 2.2. Protopolybia-MP III Blocks Multiple Steps of the HSV-1 Life Cycle

**Figure 3:**
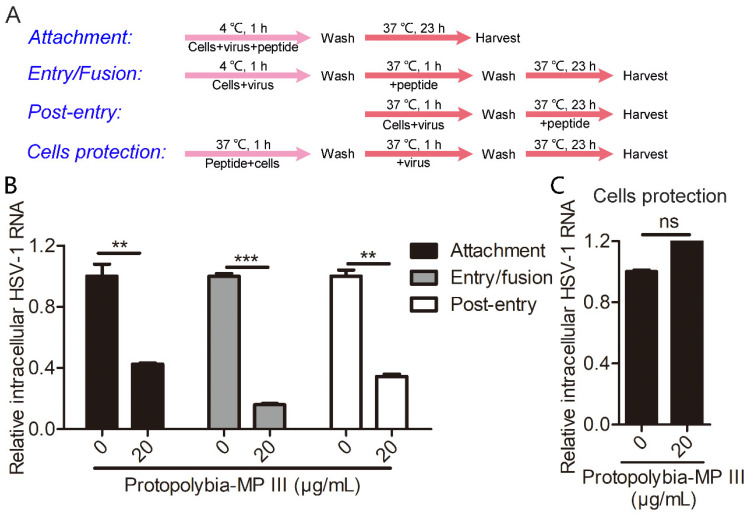
Protopolybia-MP III inhibits multiple phases of the HSV-1 life cycle. (**A**) Schematic diagram for studying stage at which Protopolybia-MP III acts on the HSV-1 life cycle. (**B**) Effects of Protopolybia-MP III on the attachment, entry/fusion, and post-entry stages of the HSV-1 life cycle. Vero cells were infected with HSV-1 at an MOI of 1 and treated with 20 μg/mL Protopolybia-MP III as described in (**A**). After 24 h, cells were collected, and intracellular HSV-1 RNA was analyzed by qPCR. (**C**) The effect of Protopolybia-MP III on cell protection against HSV-1 infection. Vero cells were treated with 20 μg/mL Protopolybia-MP III or PBS and infected with HSV-1 at an MOI of 1 as described in (**A**). After 24 h, the cells were collected, and intracellular HSV-1 RNA was analyzed by qPCR. ns, no significance. ** *p* < 0.01. *** *p* < 0.001.

To determine the exact step at which Protopolybia-MP III blocks HSV-1 infection, we conducted an experiment with different modes of peptide treatment in Vero cells (Figure 3A). The experimental results showed that 20 μg/mL Protopolybia-MP III inhibited the attachment, entry/fusion, and post-entry stages of the HSV-1 life cycle with inhibitory rates of 57%, 84%, and 66%, respectively (Figure 3B). Then, we detected the protective effect of Protopolybia-MP III by pretreating Vero cells with 20 μg/mL Protopolybia-MP III before HSV-1 infection. The results showed that Protopolybia-MP III had almost no effect on protecting cells from HSV-1 infection (Figure 3C). These results suggest that the wasp venom peptide Protopolybia-MP III has inhibitory effects on multiple stages of the HSV-1 life cycle.

**Figure 4:**
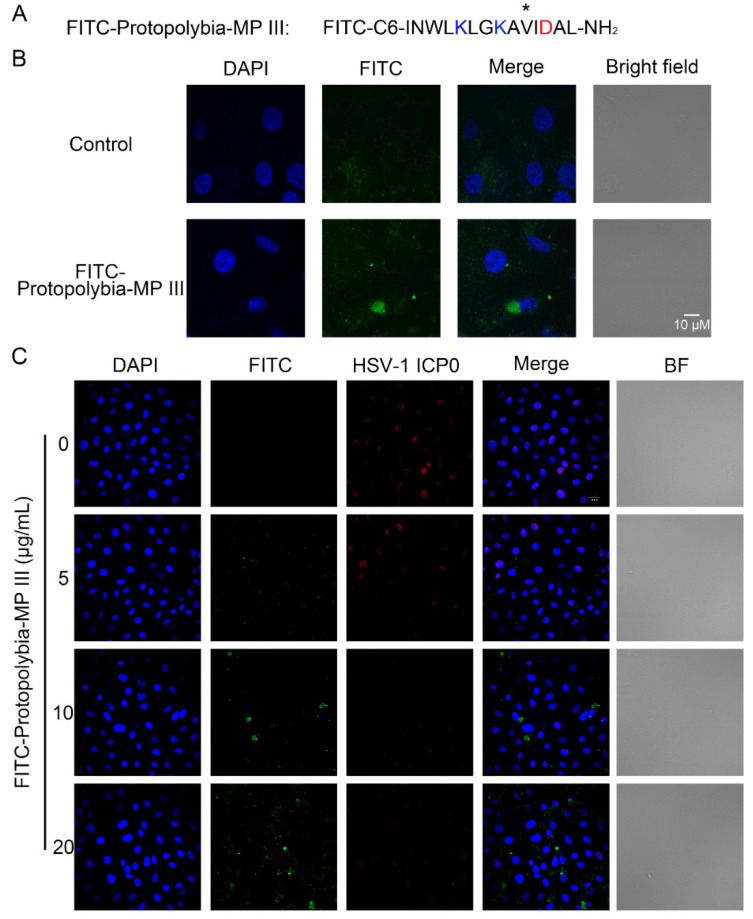
FITC-Protopolybia-MP III can enter cells and inhibit HSV-1 replication. (**A**) Amino acid sequence of FITC-Protopolybia-MP III. The tenth amino acid is labeled with *. (**B**) The distribution of FITC-Protopolybia-MP III in Vero cells. Cells were treated with FITC-Protopolybia-MP III at different concentrations or PBS for 24 h, and then cells were observed using confocal microscopy. (**C**) The inhibitory effect of FITC-Protopolybia-MP III on HSV-1 replication in Vero cells. Cells were preincubated with FITC-Protopolybia-MP III at different concentrations or PBS for 1 h and then infected with HSV-1 at an MOI of 1. After 24 h, the cells were observed using confocal microscopy. FITC, blue. HSV-1 ICP0, red. DAPI, green.

Based on these interesting experimental results, we examined the distribution of Protopolybia-MP III in Vero cells. First, FITC-Protopolybia-MP III, whose amino terminus carries a FITC label, was synthesized (Figure 4A). Then, green fluorescence was observed in the cytoplasm of Vero cells incubated with FITC-Protopolybia-MP III, while no green fluorescence was observed in the control cells treated with PBS by immunofluorescence. It revealed that FITC-Protopolybia-MP III could enter Vero cells and was mainly distributed in the cytoplasm (Figure 4B). Next, the antiviral activity of FITC-Protopolybia-MP III was further examined by immunofluorescence. It was observed that after treating HSV-1-infected Vero cells with FITC-Protopolybia-MP III, red fluorescence decreased with the increase of green fluorescence. The results showed that the more FITC-Protopolybia-MP III in Vero cells, the lower the intracellular HSV-1 ICP0 protein content (Figure 4C). In short, these results indicate that the wasp venom peptide Protopolybia-MP III can enter cells and has inhibitory effects on multiple stages of the HSV-1 life cycle.

### 2.3. Protopolybia-MP III Destroys the Integrity of the HSV-1 Virion

**Figure 5:**
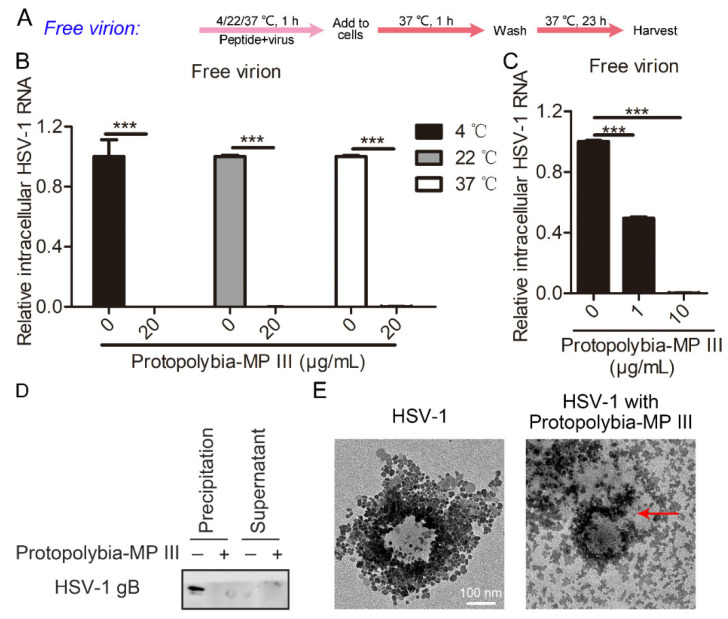
Protopolybia-MP III destroys the integrity of HSV-1 free virions. (**A**) Schematic diagram for studying the effect of Protopolybia-MP III on HSV-1 free virion infectivity. (**B**) Effects of Protopolybia-MP III on HSV-1 free virion infectivity at different temperatures. HSV-1 (MOI = 1) and 20 μg/mL Protopolybia-MP III or PBS were coincubated at 4 °C, 22 °C, and 37 °C for 1 h, and then Vero cells were infected as described in (**A**). After 24 h, cells were collected, and intracellular HSV-1 RNA was analyzed by qPCR. (**C**) Effects of different concentrations of Protopolybia-MP III on HSV-1 free virion infectivity. HSV-1 (MOI = 1) and 1 or 10 μg/mL Protopolybia-MP III or PBS were coincubated at 37 °C for 1 h, and then Vero cells were infected as described in (**A**). After 24 h, cells were collected, and intracellular HSV-1 RNA was analyzed by qPCR. (**D**,**E**) The effect of Protopolybia-MP III on HSV-1 free virion integrity. HSV-1 and 20 μg/mL Protopolybia-MP III or PBS were coincubated at 37 °C for 1 h. Then, after centrifuging for 2 h at 40,000 rpm at 4 °C, the contents of HSV-1 gB in the supernatant and precipitate were detected by Western blotting (**D**). In addition, the structure of HSV-1 virions after incubation with Protopolybia-MP III was observed by electron microscopy. The red arrow points to a destroyed HSV-1 virion. *** *p* < 0.001.

Antiviral peptides have various mechanisms of action; they may exert antiviral activity by inhibiting the viral replication cycle, or they may directly inactivate the virion, as described in the Introduction. Therefore, we analyzed the effects of Protopolybia-MP III on the free virion infectivity of HSV-1 at different temperatures (Figure 5A). The results showed that 20 μg/mL Protopolybia-MP III significantly inhibited HSV-1 virion infectivity at 4 °C, 22 °C, and 37 °C, with inhibition rates above 99% (Figure 5B). These results indicated that Protopolybia-MP III inactivated HSV-1 virions at different temperatures. Next, we analyzed the effects of Protopolybia-MP III on the free virion infectivity of HSV-1 at different concentrations, and the results showed that 1 and 10 μg/mL Protopolybia-MP III inactivated 51% and 99% of HSV-1 virions at 37 °C, respectively (Figure 5C). To further analyze the mechanism by which Protopolybia-MP III inhibited the infectivity of HSV-1 virions, HSV-1 protein content was analyzed after coincubation of HSV-1 virions with Protopolybia-MP III. HSV-1 glycoprotein gB was present in the supernatant, and the precipitate after coincubation of HSV-1 virions and Protopolybia-MP III was separated by ultracentrifugation and detected by Western blotting assay. The experimental results showed that the gB content in the precipitate after treatment with Protopolybia-MP III was significantly reduced compared with PBS treatment, while the gB content in the supernatant increased (Figure 5D). Next, to further confirm the destruction of HSV-1 virion integrity by Protopolybia-MP III, Protopolybia-MP III was incubated with HSV-1 virions and treated with negative staining, and HSV-1 virion morphology was observed by electron microscopy. We observed that the HSV-1 virion structure was destroyed after treatment with Protopolybia-MP III (Figure 5E). In summary, these results indicate that Protopolybia-MP III disrupts HSV-1 virion integrity to restrict viral infection.

### 2.4. Protopolybia-MP I Is a Natural Homolog of Protopolybia-MP III with Weak Antiviral Activity against HSV-1

**Figure 6:**
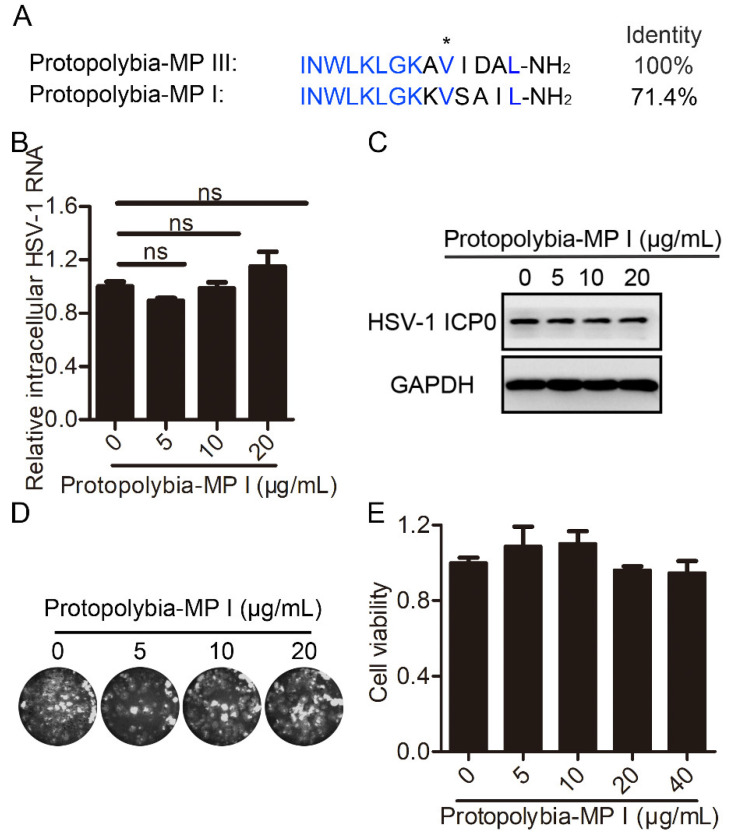
Protopolybia-MP I slightly inhibits HSV-1 replication in Vero cells. (**A**) Amino acid sequence alignment of Protopolybia-MP I and Protopolybia-MP III. The identical amino acids of Protopolybia-MP I and Protopolybia-MP III are marked in blue. (**B**,**C**) The effect of Protopolybia-MP I on intracellular HSV-1 content. Cells were preincubated with Protopolybia-MP I at different concentrations or PBS for 1 h and then infected with HSV-1 at an MOI of 1. After 24 h, the cells were collected. Intracellular HSV-1 RNA was analyzed by qPCR (**B**), and intracellular HSV-1 ICP0 protein levels were analyzed by Western blotting (**C**). (**D**) The effect of Protopolybia-MP I on extracellular HSV-1 particles. Cells were treated with Protopolybia-MP I at different concentrations or PBS for 24 h, and then the supernatant was collected and evaluated in a plaque formation assay. (**E**) Cytotoxicity of Protopolybia-MP I to Vero cells. Cells were treated with Protopolybia-MP I at different concentrations for 24 h, and then cytotoxicity was examined by the CCK8 assay. * *p* < 0.05.

Protopolybia-MP I and Protopolybia-MP III are derived from the same wasp venom, and Protopolybia-MP I has four amino acids different from those of Protopolybia-MP III (Figure 6A). Therefore, we further analyzed the antiviral activity of Protopolybia-MP I against HSV-1. At Protopolybia-MP I concentrations of 5, 10, and 20 μg/mL, Protopolybia-MP I had no effect on intracellular HSV-1 RNA and slightly inhibited HSV-1 ICP0 expression levels, with inhibition rates of 17%, 17%, and 25%, respectively, as calculated by ImageJ software (Figure 6B,C). Plaque-forming assays showed that Protopolybia-MP I had almost no inhibitory effect on extracellular HSV-1 virions (Figure 6D). Analogously, the cytotoxicity of Protopolybia-MP I to Vero cells was detected by a CCK8 assay, and we found that Protopolybia-MP I at 40 μg/mL was almost nontoxic (Figure 6E). Overall, these results indicate that Protopolybia-MP I has almost no inhibition of HSV-1 replication under conditions of no cytotoxicity.

### 2.5. Mutant Peptide Design and Antiviral Activity Analysis Based on Two Natural Wasp Venom Peptides Protopolybia-MP III and Protopolybia-MP I

**Figure 7:**
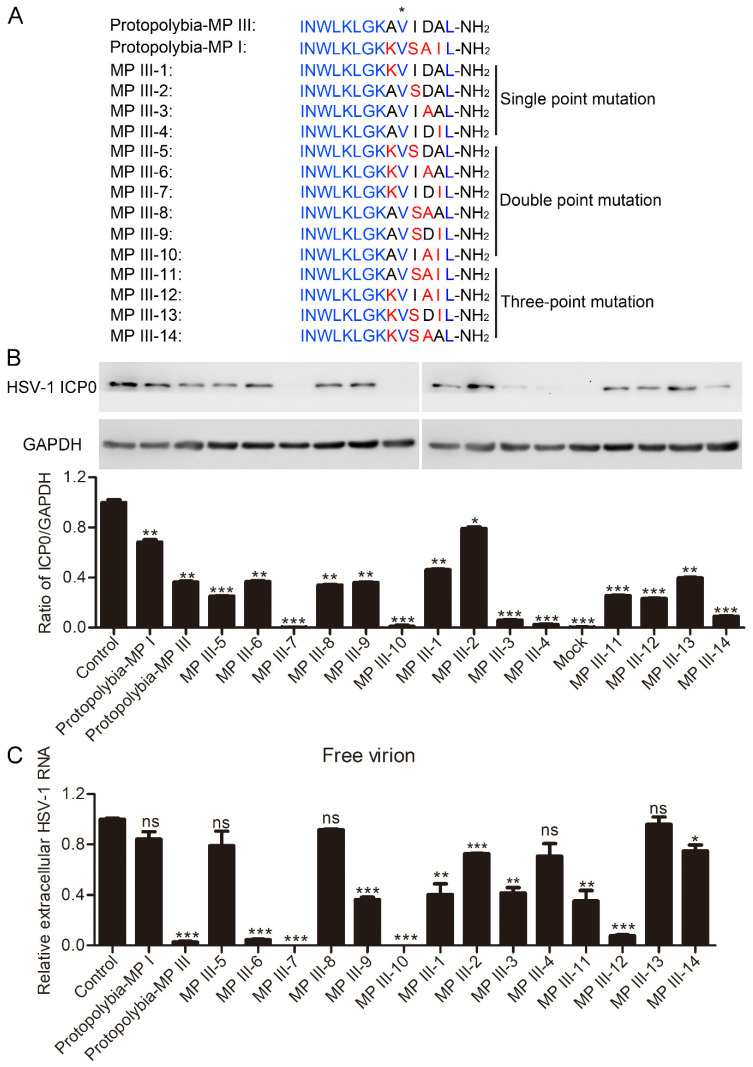
Antiviral activity analysis of Protopolybia-MP III mutants. (**A**) Amino acid sequences of Protopolybia-MP III mutants. Identical amino acids among Protopolybia-MP I, Protopolybia-MP III, and its mutants are marked in blue, and different amino acids are marked in red. (**B**) Suppression of Protopolybia-MP I, Protopolybia-MP III, and its mutants on intracellular HSV-1 content. Cells were preincubated with 20 μg/mL peptides or PBS for 1 h and then infected with HSV-1 at an MOI of 1. After 24 h, the cells were collected. Intracellular HSV-1 ICP0 protein levels were analyzed by Western blotting. The ratios of HSV-1 ICP0 to GAPDH were calculated by ImageJ software. (**C**) Inhibition of HSV-1 free virion infectivity by Protopolybia-MP I, Protopolybia-MP III, and its mutants. HSV-1 (MOI = 1) and 5 μg/mL peptides or PBS were coincubated at 37 °C for 1 h, and the mixtures were then used to infect Vero cells. After 24 h, cells were collected, and intracellular HSV-1 RNA was analyzed by qPCR. ns, no significance. * *p* < 0.05. ** *p* < 0.01. *** *p* < 0.001.

Protopolybia-MP III has significantly higher anti-HSV-1 activity than Protopolybia-MP I but differs from Protopolybia-MP I in its amino acid sequence by only four amino acids. Therefore, we analyzed the anti-HSV-1 activity of 14 Protopolybia-MP III mutants previously designed in our laboratory according to the sequences of Protopolybia-MP III and Protopolybia-MP I [28]. Four are single-point mutations of Protopolybia-MP III named MP III-1/2/3/4, six are double-point mutations named MP III-5/6/7/8/9/10, and four are three-point mutations named MP III-11/12/13/14 (Figure 7A). Western blotting assay revealed that the inhibition rates of 20 μg/mL Protopolybia-MP I and Protopolybia-MP III, MP III-5/6/7/8/9/10/1/2/3/4/11/12/13/14 on intracellular HSV-1 ICP0 protein expression were 31.5%, 63.5%, 74.8%, 63.2%, 100%, 66%, 63.8%, 100%, 53.7%, 20.6%, 94%, 99.4%, 73.4%, 76.8%, 60.2%, and 90.8%, respectively, as calculated by ImageJ software (Figure 7B). These results demonstrated that the antiviral activity of MP III-2 was significantly lower than that of Protopolybia-MP III, which indicated that the Ile_11_ residue of Protopolybia-MP III plays an important role in antiviral function. Surprisingly, the antiviral activity levels of MP III-3/4/7/10/14 were significantly higher than those of Protopolybia-MP III. Furthermore, we analyzed the effects of Protopolybia-MP I, Protopolybia-MP III, and their mutants on the free virion infectivity of HSV-1. qPCR assay showed that 5 μg/mL Protopolybia-MP III and MP III-6/7/9/10/1/2/3/11/12/14 significantly inactivated HSV-1-free virions at 37 °C, with inhibition rates of 97.3%, 95.5%, 100%, 63.6%, 100%, 59.7%, 27.4%, 58.3%, 64.7%, 92.4%, and 25.3%, respectively (Figure 7C). Overall, MP III-7 and MP III-10 showed excellent antiviral activity against HSV-1 and are promising antiviral peptide drug candidates.

## 3. Discussion

Viral infection is a serious threat to human health and is commonly treated with antiviral drugs in clinical practice, which mainly include organic small molecules, active peptides, monoclonal antibodies, and other biological agents [2]. Importantly, active AVPs have the advantages of high efficiency, high specificity, and good safety [3,29,30]. At present, the AVP database (http://crdd.osdd.net/servers/avpdb) (accessed on 29 December 2023) contains 2683 targeted AVPs related to more than 60 antiviral medicines that have great potential to become antiviral drugs [6]. Mastoparan family peptides derived from wasp venom exert various pharmacological effects, including bacteriostasis, immune regulation, and antitumor effects [16,17,18]. However, the antiviral function of natural wasp venom peptides has not been reported.

In our study, the antiviral activity of the natural wasp venom peptide Protopolybia-MP III was identified for the first time. The results showed that micromolar levels of Protopolybia-MP III strongly suppressed HSV-1 replication in multiple cell lines, including HSV-1-susceptible Vero cells and the human cell lines A549, HCEC, and SK-N-MC (Figure 1 and Figure 2). It is worth noting that the primary infection sites of HSV-1 mainly include epithelial tissues, while sensory neurons represent the main sites of the latent infection [20]. Therefore, the anti-HSV-1 activities of Protopolybia-MP III in HCEC and SK-N-MC are very meaningful because they are expected to inhibit latent HSV-1 infection, which is worth analyzing in the future. Next, to determine the antiviral mechanism of Protopolybia-MP III, its effects on the HSV-1 life cycle and its free virion infectivity were examined. The data showed that Protopolybia-MP III not only inhibited the attachment, viral entry/fusion, and post-entry stages of the HSV-1 life cycle but also significantly suppressed HSV-1-free virion infectivity (Figure 3 and Figure 5). More importantly, the antiviral activity of Protopolybia-MP III against the latter was higher than that of the former. Therefore, the mechanism by which Protopolybia-MP III inhibits virion infectivity was analyzed by ultracentrifugation and electron microscopy. We found that Protopolybia-MP III disrupted HSV-1 virion integrity to inactivate HSV-1. In addition, because Protopolybia-MP III also had an inhibitory effect on multiple stages of the HSV-1 replication cycle, we synthesized FITC-Protopolybia-MP III and determined its distribution in Vero cells. The results of immunofluorescence analysis revealed that FITC-Protopolybia-MP III decreased HSV-1 ICP0 expression levels in Vero cells, further confirming its antiviral effect in vitro (Figure 4). Many existing AVPs only inhibit virion infection or a certain phase of the viral replication cycle [9,31]. There are few AVPs that both restrict multiple stages of the viral replication cycle and destroy the integrity of the virion [32,33,34]. Among the common antiviral drugs, the antiviral mechanisms of ribavirin include both indirect mechanisms (inosine monophosphate dehydrogenase inhibition, immunomodulatory effects) and direct mechanisms (interference with RNA capping, polymerase inhibition, lethal mutagenesis); ACR inhibits the synthesis of viral nucleic acids; amantadine and rimantadine inhibit viral replication through the induction of autophagy; and interferons and polyinosinic-polycytidylic acid can play antiviral roles by boosting immunity [23,35,36,37]. Accordingly, the identification of AVP Protopolybia-MP III and the elucidation of its antiviral mechanism are of great significance. However, the antiviral effect of these wasp venom peptides *in vivo* needs to be analyzed in the future.

In conclusion, our study identified a new natural AVP, Protopolybia-MP III, and determined its antiviral action stages and the mechanism by which Protopolybia-MP III mainly disrupted HSV-1 virion integrity to inactivate virions. In addition, we screened several optimized mutants of Protopolybia-MP III with higher antiviral activity, suggesting that the Protopolybia-MP III-containing Mastoparan peptide family might represent a new molecular scaffold against viruses. These works provide new ideas for the medicinal use of peptides in wasp venom and lay a foundation for the research and development of antiviral candidate peptide drugs.

## 4. Materials and Methods

### 4.1. Peptide Synthesis and Purification

The peptides were chemically synthesized from the C-terminus to the N-terminus according to their amino acid sequence using the solid-phase synthesis method with a purity of >95%, and amidation reactions were performed at the C-termini of all peptides (ChinaPeptides Co., Ltd., Shanghai, China). A FITC tag was added at the N-terminus of the peptide Protopolybia-MP III (Chinese Peptide Company, Hangzhou, China). The synthetic peptides were assessed by high-performance liquid chromatography (HPLC) and mass spectrometry (Voyager-DESTR; Applied Biosystems, Waltham, MA, USA). Peptides were dissolved in PBS at a final concentration of 1 mg/mL for subsequent testing, and PBS was used as a negative control.

### 4.2. Cell Culture and Virus Infection

Vero and A549 cells were provided by Professor Zhijian Cao of Wuhan University. HCEC and SK-N-MC were purchased from the Bena Culture Collection (Beijing, China). All cells were cultured in Dulbecco’s modified Eagle’s medium (Gibco-Invitrogen, New York, NY, USA) supplemented with 10% fetal bovine serum (FBS) (Gibco-Invitrogen), 1% penicillin, and 1% streptomycin (Gibco-Invitrogen) at 37 °C in a 5% CO_2_ incubator.

HSV-1 was kindly provided by Professor Zhijian Cao of Wuhan University. Cells were infected with HSV-1 at an MOI of 1 for 24 h. Then, cells were collected for qPCR, Western blotting, and immunofluorescence assays, and the extracellular supernatant was used for plaque formation assays.

### 4.3. CCK8 Assay

Cells were seeded in a 96-well plate (7000–10,000 cells per well) and cultured at 37 °C overnight. Peptides in a series of concentrations were added to the medium, and cells were then incubated at 37 °C for 24 h. Then, 10 μL CCK8 solution (5 mg/mL in PBS buffer; Invitrogen) and 90 μL medium were used to incubate cells at 37 °C for 2 h. Finally, the absorbance was measured at 450 nm using a microplate reader (BioTek, Winooski, VT, USA).

### 4.4. qPCR

Cells were collected and lysed using TRIzol reagent (Beyotime Biotechnology, Shanghai, China) to release the intracellular total RNA, followed by precipitation with isopropanol. The first-strand cDNA was reverse-transcribed by the HiScript III 1st Strand cDNA Synthesis Kit (+gDNA wiper) (Vazyme, Nanjing, China). The cDNA was quantitated by qPCR with primers using Taq Pro Universal SYBR qPCR Master Mix (Vazyme). The HSV-1 primers were 5′-ATGGGAGGCAACTGTGCTAT-3′ (sense) and 5′-CTCGGTGCTCCAGGATAAAC-3′ (antisense). The monkey GAPDH primers were 5′-TCAACGACCACTTTGTCAAGCTCA-3′ (sense) and 5′-GCTGGTGGTCCAGGGGTCTTACT-3′ (antisense). The human GAPDH primers were 5′-TGATGACATCAAGAAGGTGGTGAAG-3′ (sense) and 5′-TCCTTGGAGGCCATGTGGGCCAT-3′ (antisense). qPCR experiments were performed on a Bio-Rad CFX96 Touch (Pleasanton, CA, USA) according to the manufacturer’s instructions.

### 4.5. Western Blotting

Cells were collected and lysed in 1% SDS. The cell lysate was heated at 100 °C for 20 min and centrifuged at 12,000 rpm at 4 °C for 15 min, and then the supernatant was collected and assayed with a BCA Protein Quantification Kit (Vazyme). Equal amounts of protein were separated using a PAGE Gel Fast Preparation Kit (10%) (Epizyme Biotech, Wuhan, China) and transferred to nitrocellulose (NC) membranes (Millipore, Wuhan, China). The nonspecific proteins on the NC membranes were blocked with 5% skim milk, and the membranes were incubated on a shaker for 2 h at room temperature. The primary antibodies were then incubated overnight at 4 °C, and the secondary antibody was incubated for 2 h at room temperature. The results were analyzed using a Clarity Western ECL Substrate (Bio-Rad) with a ChemiDoc Chemiluminescent Gel Imaging System (Bio-Rad). The primary antibodies were mouse monoclonal anti-HSV-1 ICP0 (sc-53070) (Santa Cruz Biotechnology, Paso Robles, CA, USA), mouse monoclonal anti-HSV-1/2 gB (sc-56987) (Santa Cruz Biotechnology), and mouse monoclonal anti-GAPDH (60004-1-Ig) (Proteintech group, Rosemont, IL, USA).

### 4.6. Plaque Formation Assay

Vero cells seeded in a 24-well plate at 100% confluence were infected with virus diluent or supernatant. After 1 h of absorption at 37 °C, the supernatant was removed. Cells were washed three times with PBS and replenished with a plaque-covering layer (MEM with 2% FBS and 1.5% carboxymethylcellulose). After an incubation period of approximately 3 days, the cells were stained with 1% crystal violet containing 10% methanol for 0.5 h. Then, the plaques were counted, and the viral titer was calculated according to the plaque number and dilution.

### 4.7. Immunofluorescence Assay

Cells were fixed with precooled 4% paraformaldehyde for 10 min and permeabilized with 0.2% Triton X-100 for 5 min. Then, nonspecific proteins were blocked with 5% bovine serum albumin (BSA) for 1 h at room temperature, followed by incubation with 1% BSA-diluted mouse monoclonal anti-HSV-1 ICP0 overnight at 4 °C. Subsequently, the cells were incubated with 1% BSA-diluted Alexa Fluor 647 AffiniPure Donkey Anti-Mouse IgG (H + L) (34113ES60) (YEASEN, Shanghai, China) for 1 h at room temperature in the dark. Then, the nuclei were stained with DAPI (ANT10072) (Epizyme Biotech). The fluorescence was observed using a confocal laser-scanning microscope with ultrahigh resolution (FV3000RS) (Olympus Corporation, Tokyo, Japan).

### 4.8. Virion Integrity Assay

HSV-1 virions were coincubated with PBS or 20 μg/mL Protopolybia-MP III at 37 °C for 1 h. Then, the virions were separated by ultracentrifugation at 40,000 rpm at 4 °C for 2 h in a Beckman SW41 rotor (Beckman Coulter, Brea, CA, USA). The supernatant was collected and lyophilized using a vacuum freeze dryer (Labconco FreeZone6L, Kansas City, MO, USA). HSV-1 glycoprotein gB from both the precipitate and supernatant components were measured by Western blotting. In addition, after HSV-1 was coincubated with PBS or 20 μg/mL Protopolybia-MP III at 37 °C for 1 h, samples were placed on 200-mesh var/carbon-coated copper grids by dipping the grids into the virion sample solutions. Each sample was then negatively stained by placing a drop of 2% phosphotungstic acid (pH = 7, adjusted with NaOH) on the grid. The sample grid was then air-dried overnight. Finally, various areas of the grid were examined, and photographs were taken with a HITACHI H-8100 transmission electron microscope operating at 100 kV.

### 4.9. Statistical Analysis

Adobe Photoshop CC (64 Bit), ImageJ 1.52a, Image Lab 5.2, and Graphpad Prism 5 were used for statistical analysis. Data represent the mean ± standard deviation (SD) of at least three independent experiments, and *p* values were calculated by T.TEST. Statistical significance was considered at a *p* value less than 0.05 (* *p* < 0.05. ** *p* < 0.01. *** *p* < 0.001. ns, no significance).

## Data Availability

All data supporting the results can be found within the manuscript.
